# A Case of Statin-Induced Fulminant Hepatitis Presenting as Septic Shock and Multiorgan Failure

**DOI:** 10.7759/cureus.114371

**Published:** 2026-08-11

**Authors:** Matthew E Lehrack, John Daliva, Nadir E Eltahir, Ronald Chang, Aftab Qadir

**Affiliations:** 1 Internal Medicine, Western University of Health Sciences, Pomona, USA; 2 Internal Medicine, Pomona Valley Hospital Medical Center, Pomona, USA; 3 Intensive Care Medicine, Pomona Valley Hospital Medical Center, Pomona, USA

**Keywords:** statin-induced liver necrosis, statin overdose, statin safety, statin side effects, statin toxicity

## Abstract

Statins are among the most widely prescribed medications for hyperlipidemia, with drug-induced liver injury (DILI) being a rare adverse event. Here, we report a case involving a 69-year-old female patient who developed fulminant liver failure following an overdose of rosuvastatin. The patient had ingested 60 tablets of rosuvastatin 20 mg over the one-week period prior to hospital admission, mistakenly believing it was her pain medication. This acute high-dose exposure far exceeded recommended dosing and resulted in fulminant hepatitis and multiorgan failure. Despite comprehensive critical care, including advanced supportive and rescue interventions, the patient’s condition deteriorated, and she did not survive. This underscores the potential lethality of statin overdose and the importance of medication safety, early recognition, and prevention strategies.

## Introduction

Rosuvastatin, marketed as Crestor, is a widely prescribed 3-hydroxy-3-methylglutaryl coenzyme A (HMG-CoA) reductase inhibitor indicated for lowering low-density lipoprotein (LDL) cholesterol [1,2]. When prescribed in higher doses (20-40 mg), it may be used as moderate or high-intensity statin therapy for atherosclerosis and the reduction of major adverse cardiovascular events. It is generally well tolerated, with the most frequent adverse reactions (≥2%) including myalgias, headache, nausea, asthenia, constipation, and arthralgias [1]. It has similar rates of elevated transaminases compared to other statins, with some studies suggesting severely elevated transaminases (>3 times the upper limit of normal) occur no more frequently than placebo [2]. Risk factors for severe muscle injury and hepatic events include age ≥65 years, high-dose therapy, renal impairment, and concomitant use of interacting medications [3,4]. Large pharmacovigilance studies and registry data confirm that all statins are associated with drug-induced liver injury (DILI), with the highest reporting rates for fluvastatin, atorvastatin, and simvastatin, with rosuvastatin accounting for approximately 20% of cases [5-7].

The risk of severe statin-induced liver injury at therapeutic doses, including acute liver failure, is extremely low, estimated at approximately one in 100,000 treated individuals [6-8]. These mechanisms are typically idiosyncratic, with none being attributed to statin overdose. Clinical trials suggest that accidental or intentional statin overdose can dramatically increase the risk of severe hepatotoxicity, but published case reports of statin overdose contributing to hepatic failure remain scarce [5-9].

## Case presentation

Initial presentation

A 69-year-old woman who was a Jehovah’s Witness, with a history of hypertension, prediabetes, cervical spinal stenosis, beta-thalassemia trait, supraventricular tachycardia, osteoporosis, major depressive disorder, and a reported urea cycle metabolism disorder, presented to the emergency department of a community hospital with a three-day history of dyspnea, intractable nausea, vomiting, diarrhea, bilateral carpopedal spasms, and severe muscle cramping.

Seven days prior, the patient was evaluated by her primary care physician for knee pain and prescribed acetaminophen. She also had 20 mg rosuvastatin refilled at that time. Due to a medication error, she was taking rosuvastatin multiple times per day instead, believing it was her pain medication. The patient consumed approximately 60 tablets over one week. She denied alcohol use and had a remote tobacco use history.

On arrival, she was hypotensive with systolic blood pressure in the 80-90 mmHg range, tachycardic in the 110-120 range, and tachypneic, with respiratory rate between 20 and 29 breaths/min. On physical examination, the patient was alert and oriented ×3 with a Glasgow Coma Scale of 15. Skin examination revealed no jaundice. Abdominal exam demonstrated diffuse tenderness without rebound or guarding. Deep tendon reflexes were brisk (3+) bilaterally. Carpopedal spasms were present in the upper and lower extremities. 

The patient's clinical presentation required aggressive resuscitation with fluids and vasopressor support, as well as intubation. Initial management included intravenous (IV) fluids, empiric vancomycin and piperacillin-tazobactam, albumin, calcium gluconate, intravenous steroids, and mechanical ventilation. Initial laboratory results are provided in Tables 1, 2.

**Table 1:** Admission liver function tests (LFTs) and international normalized ratio (INR). Admission liver chemistries demonstrated a predominantly hepatocellular pattern of injury with mild transaminitis and early impairment of hepatic synthetic function (elevated INR) in the absence of hyperbilirubinemia. Although values were only modestly elevated at presentation, these findings preceded the rapid progression to fulminant hepatic failure observed later in the hospital course.

Laboratory tests	Patient values	Reference range
Aspartate aminotransferase (AST)	262 U/L	10-40 U/L
Alanine aminotransferase (ALT)	104 U/L	7-56 U/L
Alkaline phosphatase (ALP)	218 U/L	40-147 U/L
Total bilirubin	0.4 mg/dL	0.2-1.2 mg/dL
International normalized ratio (INR)	1.4	0.9-1.1

**Table 2:** Other abnormal laboratory studies on admission. Admission laboratory studies demonstrated severe multisystem derangements including leukocytosis, anemia, profound electrolyte abnormalities, high anion gap metabolic acidosis, and severe acute kidney injury. Elevated lactate and procalcitonin were suggestive of a diagnosis of shock with systemic inflammatory response, while renal failure and metabolic acidosis suggested early multiorgan dysfunction preceding overt hepatic failure. The bilateral carpopedal spasms noted on presentation were consistent with the patient's severe hypocalcemia (4.5 mg/dL), a recognized manifestation of both acute renal failure and rhabdomyolysis-associated calcium sequestration into injured muscle. No baseline creatinine was available for comparison; the reported eGFR of 5 mL/min/1.73 m² (calculated via the CKD-EPI equation) reflects the patient's renal function at presentation and cannot definitively distinguish acute kidney injury from acute-on-chronic disease in the absence of prior records. eGFR: estimated glomerular filtration rate; CKD-EPI: Chronic Kidney Disease Epidemiology Collaboration

Laboratory tests	Patient values	Reference range
White blood cell count	23.5×10⁹/L	4.0-11.0×10⁹/L
Hemoglobin	8.1 g/dL	12.0-16.0 g/dL
Sodium	122 mmol/L	135-145 mmol/L
Potassium	3.1 mmol/L	3.5-5.0 mmol/L
Chloride	83 mmol/L	98-107 mmol/L
Bicarbonate	10 mmol/L	22-28 mmol/L
Anion gap	29 mEq/L	8-16 mEq/L
Blood urea nitrogen	67 mg/dL	7-20 mg/dL
Creatinine	8.0 mg/dL	0.6-1.3 mg/dL
eGFR	5 mL/min/1.73 m²	>60 mL/min/1.73 m²
Calcium (total)	4.5 mg/dL	8.6-10.2 mg/dL
Troponin	132-152 ng/L	<14 ng/L
Lactate	3.5 mmol/L	0.5-2.2 mmol/L
Procalcitonin	2.04 ng/mL	<0.1 ng/mL

Hepatitis panel, ethanol level, salicylate level, and urinalysis were negative on admission. Acetaminophen level was 14 µg/mL initially. Given the acetaminophen prescription for knee pain one week prior, this level was felt to be most consistent with recent therapeutic dosing rather than chronic supratherapeutic use. The exact time since last acetaminophen dose relative to the initial level could not be precisely established, limiting formal placement on the Rumack-Matthew nomogram. The patient did not describe deviation from the prescribed acetaminophen regimen, and there was no clinical or biochemical pattern (such as a disproportionate alanine aminotransferase {ALT} rise relative to aspartate aminotransferase {AST}, or a level trending upward) to suggest overlapping acetaminophen-induced injury. Over the preceding days, the acetaminophen level trended downward to 5 µg/mL without a rising or supratherapeutic trajectory. Given these findings, acetaminophen toxicity was considered unlikely, though it could not be definitively excluded on nomogram grounds alone [10]. Imaging demonstrated pulmonary edema and hepatic steatosis without biliary obstruction, but otherwise no acute hepatic findings. The patient was admitted with presumed septic shock and acute kidney injury vs. acute kidney injury on top of chronic kidney disease.

Hospital course

Despite broad-spectrum antibiotics and vasopressor support, the patient remained critically ill. Continuous renal replacement therapy (CRRT) was initiated on hospital day three for severe acute tubular necrosis, attributed to shock and statin-induced rhabdomyolysis. Myoglobinuria from statin-induced rhabdomyolysis (peak creatine kinase {CK}: 3,542 U/L) is a recognized precipitant of acute tubular necrosis via direct tubular toxicity, intraluminal cast formation, and renal vasoconstriction [11]. This mechanism likely acted alongside shock-related hypoperfusion to drive the patient's early, severe renal failure (creatinine: 8.0 mg/dL, estimated glomerular filtration rate {eGFR}: 5 mL/min). The resulting acute kidney injury (AKI) may have further impaired rosuvastatin clearance, compounding myotoxicity, nephrotoxicity, and hepatotoxicity in a self-reinforcing cycle.

**Figure 1:**
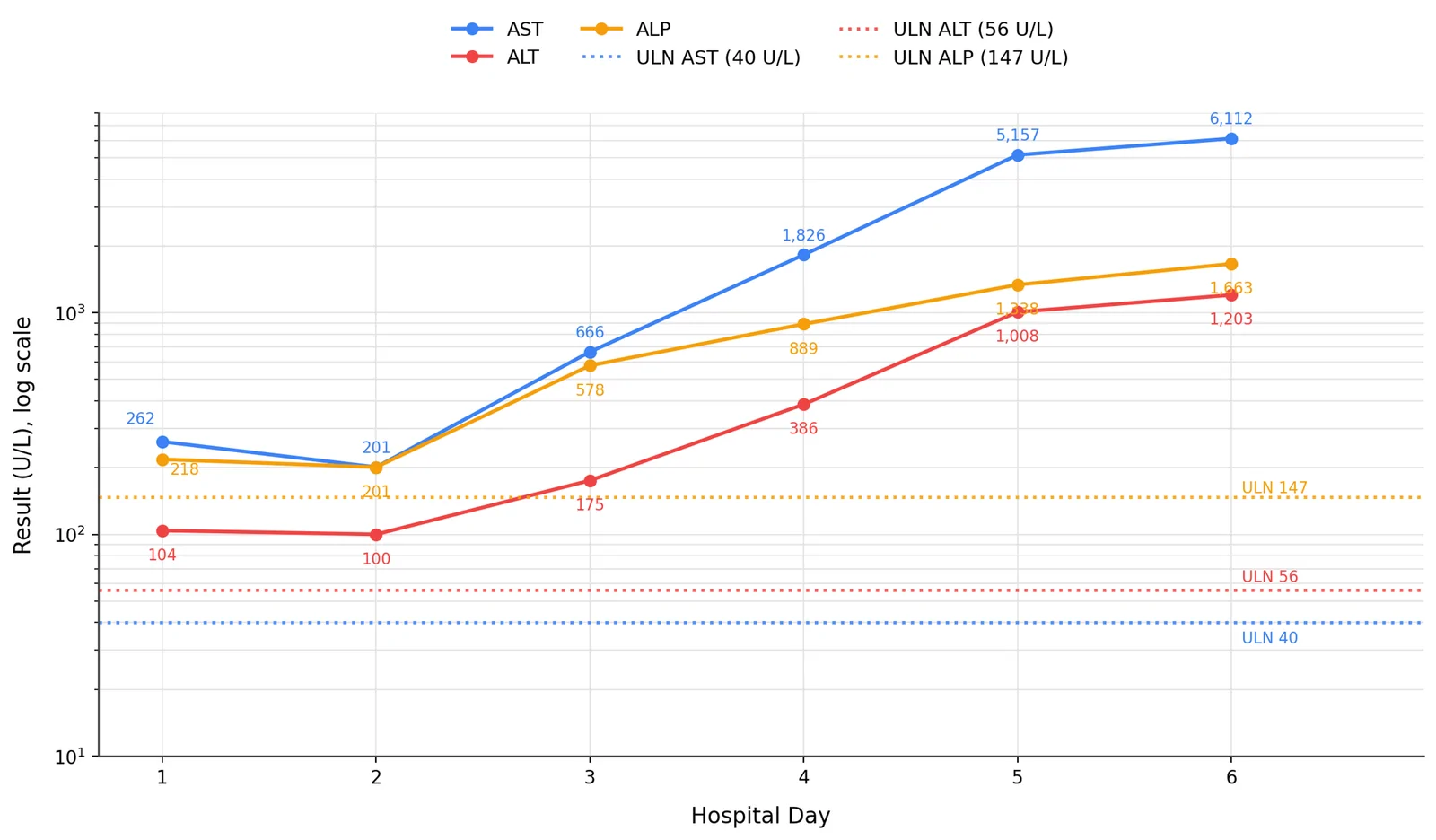
Trending of AST, ALT, and INR on a logarithmic scale. Liver function tests (LFTs) trended upward significantly to a peak AST of 6,112 U/L, ALT of 1,203 U/L, and ALP of 1,663 U/L compared to an upper limit of normal (ULN) of 40 U/L, 56 U/L, and 147 U/L, respectively. Given that AST is also abundantly expressed in skeletal muscle, the disproportionate AST elevation relative to ALT (6,112 vs. 1,203 U/L, ratio ~5:1) likely reflects a mixed contribution from both hepatocellular necrosis and ongoing rhabdomyolysis, rather than hepatic injury alone [11]. This limits the ability to use AST in isolation as a pure marker of hepatocellular injury severity in this case. AST: aspartate aminotransferase; ALT: alanine aminotransferase; ALP: alkaline phosphatase; INR: international normalized ratio

**Figure 2:**
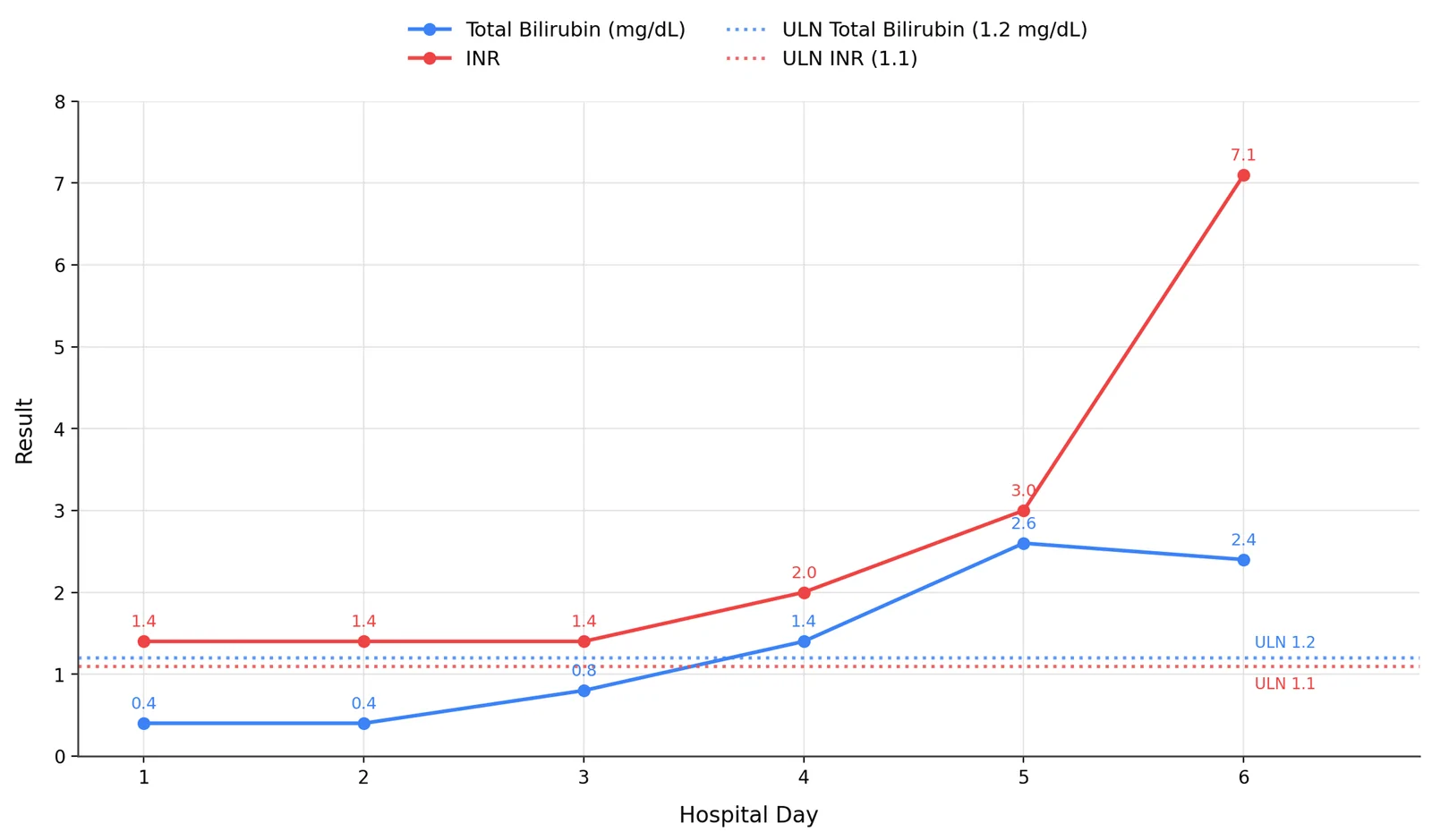
Trending total bilirubin and INR. Significant upward trends were also seen in total bilirubin and INR results, with peaks of 2.6 mg/dL and 7.1, compared to an upper limit of normal (ULN) of 1.2 mg/dL and 1.1, respectively. This further indicates catastrophic liver damage and failure in the setting of statin overdose. INR: international normalized ratio

Beginning on hospital day three, liver enzymes rose dramatically, representing a mixed picture consistent with severe rhabdomyolysis and hepatocellular injury far exceeding that expected from ischemic hepatitis alone. Poison control recommended initiation of N-acetylcysteine at this time due to signs of hepatic injury. The patient continued to receive IV steroids and vasopressors. Over subsequent days, the patient was intubated due to hypoxic respiratory failure and developed the following trends of liver enzymes and liver function tests (Figures 1, 2).

By hospital day six, the patient had developed a severe lactic acidosis (lactate: 32.7 mmol/L), elevated creatine kinase (CK) (3,542 U/L), hyperkalemia (7.1 mmol/L), and hyperammonemia (258 µmol/L) despite CRRT. She became jaundiced and profoundly hypotensive with escalating vasopressor requirements and worsening mental status. CRRT was discontinued due to hemodynamic intolerance. The Model for End-Stage Liver Disease (MELD) score at this point was calculated to be 44, suggestive of a three-month mortality >50% without a liver transplant [12]. A tertiary care center was contacted at this point and accepted the patient for liver transplant. However, the patient continued to decline and was deemed unstable for transfer to the transplant center. Goals of care were transitioned to comfort-focused measures, and she was designated do not resuscitate (DNR). The patient unfortunately died in the following hours.

## Discussion

Current literature supports the claim that statins have a generally favorable safety profile, with severe liver injury and fulminant hepatitis being extremely rare side effects. The American Heart Association reports that, after decades of statin use, only a handful of fatal cases have been reported, and the overall incidence of severe liver injury is estimated at less than one in 100,000 treated individuals [5]. The Drug-Induced Liver Injury Network (DILIN) found that among 22 cases of statin-induced DILI, four developed hepatic failure and one died [9,10]. A recent 2023 case report describes fatal hepatic failure following atorvastatin treatment, with pathological findings of focal inflammatory necrosis and exclusion of viral and autoimmune causes [13]. These existing cases were all attributed to idiosyncratic reactions at therapeutic levels, with none involving overdose of statins.

A formal causality assessment tool was not applied in this case. Objective instruments such as the Roussel Uclaf Causality Assessment Method (RUCAM) are the standard for adjudicating DILI causality. However, RUCAM was developed and validated primarily for idiosyncratic hepatotoxic reactions occurring at therapeutic dosing, incorporating criteria such as latency from therapeutic initiation and dechallenge/rechallenge patterns that do not map cleanly onto a single massive supratherapeutic overdose. In this case, the temporal relationship between an approximately 60-tablet rosuvastatin ingestion over one week and the subsequent fulminant hepatic failure was considered sufficiently direct to support causality on clinical grounds alone. Future case reports of statin overdose-associated hepatotoxicity may benefit from an adapted or overdose-specific causality framework, as existing tools were not designed for this scenario [14,15].

The interplay between severe systemic shock and direct statin-mediated hepatotoxicity likely potentiated the degree of hepatic injury observed in this case. Profound hypotension and vasopressor-refractory shock on presentation would be expected to produce a component of ischemic hepatopathy characterized by a rapid, transient transaminase rise. By contrast, the magnitude and persistence of transaminase elevation with AST exceeding 6,000 U/L by hospital day six is not typical of ischemic injury alone, which usually peaks and begins to downtrend within 24-48 h of hemodynamic stabilization. It is more plausible that hypoperfusion-mediated microvascular injury and zonal hepatocyte hypoxia lowered the threshold for, and accelerated, direct rosuvastatin-induced hepatocellular necrosis, with the two insults acting synergistically rather than independently. The combined mechanism of toxic insult superimposed on a shock liver may better explain the fulminant trajectory than either process alone [16].

The specific enzymatic defect underlying the patient's reported urea cycle metabolism disorder could not be confirmed, as records were unavailable and further collateral history could not be obtained during the rapidly deteriorating presentation. This represents a notable limitation, as an underlying urea cycle disorder could plausibly have impaired ammonia clearance independent of the acute hepatic injury, even if previously subclinical or diet-controlled. This could thereby have lowered the threshold for hyperammonemia and potentially compounded hepatic encephalopathy risk once fulminant liver failure developed. Whether this comorbidity was a meaningful contributor to the severity of this patient's course or an incidental history detail cannot be determined retrospectively, and this uncertainty should be considered when interpreting the hyperammonemia observed on hospital day six.

The patient received empiric broad-spectrum antibiotics (vancomycin and piperacillin-tazobactam) for presumed septic shock. Antibiotics are among the most common causes of idiosyncratic drug-induced liver injury and represent a relevant differential in this case [17]. However, the temporal pattern of hepatocellular injury emerging on hospital day three and progressing in parallel with a clear toxic ingestion timeline predating antibiotic initiation makes antibiotic-induced injury a less likely primary driver than rosuvastatin toxicity, though a contributory or additive effect cannot be entirely excluded.

In the case described above, the massive overdose of rosuvastatin resulted in fulminant hepatic failure, multiorgan dysfunction, and death despite aggressive critical care interventions. It represents a novel report of statin-induced liver necrosis and acute liver failure following massive statin overdose. This patient possessed risk factors such as female sex and advanced age, both of which are recognized as risk factors for statin-induced DILI [6-8,18-20].

The American Heart Association and American College of Cardiology guidelines emphasize that while routine monitoring of liver biochemistries is not required, clinicians should remain vigilant for symptoms of hepatitis or hepatic dysfunction, especially in high-risk populations or in the context of supratherapeutic dosing [5]. This case underscores the importance of medication reconciliation, patient education, and prompt recognition of statin toxicity, particularly in older adults who may be at increased risk for dosing errors and adverse outcomes.

## Conclusions

Rosuvastatin-induced liver failure is a rare but serious adverse event at therapeutic doses and is most often associated with idiosyncratic reactions. This case adds to the limited literature on fatal statin hepatotoxicity following overdose and highlights the need for ongoing vigilance, patient safety measures, and further research into the mechanisms and risk factors underlying severe statin-induced liver injury.
